# Acute severe paediatric asthma: study protocol for the development of a core outcome set, a Pediatric Emergency Reserarch Networks (PERN) study

**DOI:** 10.1186/s13063-019-3785-6

**Published:** 2020-01-13

**Authors:** Simon Craig, Franz E. Babl, Stuart R. Dalziel, Charmaine Gray, Colin Powell, Khalid Al Ansari, Mark D. Lyttle, Damian Roland, Javier Benito, Roberto Velasco, Julia Hoeffe, Diana Moldovan, Graham Thompson, Suzanne Schuh, Joseph J. Zorc, Maria Kwok, Prashant Mahajan, Michael D. Johnson, Robert Sapien, Kajal Khanna, Pedro Rino, Javier Prego, Adriana Yock, Ricardo M. Fernandes, Indumathy Santhanam, Baljit Cheema, Gene Ong, Shu-Ling Chong, Andis Graudins, On behalf of the Pediatric Emergency Research Networks (PERN), Nathan Kupperman, Stuart Dalziel, Franz Babl, Jim Chamberlain, David Johnson, Mark Lyttle, Santiago Mintegi, Rakesh Mistry, Lise Nigrovic, Amy Plint, Damian Roland, Patrick Van de Van de Voorde

**Affiliations:** 1 Paediatric Emergency Department, Monash Medical Centre, 246 Clayton Rd, Clayton, Victoria 3168 Australia; 2 Department of Paediatrics, School of Clinical Sciences at Monash Health, Monash University, Clayton, Australia; 3 Emergency Department, Royal Children’s Hospital, Melbourne, Australia; 4 Department of Paediatrics, University of Melbourne, Melbourne, Australia; 5 Murdoch Children’s Research Institute, Melbourne, Australia; 6 Paediatric Research in Emergency Departments International Collaborative (PREDICT) Network, Melbourne, Australia; 7 Starship Children’s Hospital, Auckland, New Zealand; 8 Liggins Institute, University of Auckland, Auckland, New Zealand; 9 Women’s & Children’s Hospital, Adelaide, Australia; 10 University of Adelaide, Adelaide, Australia; 11 Emergency Department, Sidra Medicine, Doha, Qatar; 12 School of Medicine, Cardiff University, Cardiff, UK; 13 Pediatric Emergency Research Qatar (PERQ) Network, ., Qatar; 14 Bristol Royal Hospital for Children, Bristol, UK; 15 Faculty of Health and Applied Sciences, University of the West of England, Bristol, UK; 16 Paediatric Emergency Research in the United Kingdom and Ireland (PERUKI), ., UK; 17 SAPPHIRE Group, Health Sciences, Leicester University, Leicester, UK; 18 Paediatric Emergency Medicine Leicester Academic (PEMLA) Group, Children’s Emergency Department, Leicester Royal Infirmary, Leicester, UK; 19 Pediatric Emergency Department, Cruces University Hospital, Bilbao, Spain; 20 Department of Pediatrics, Basque Country University, San Sebastian, Spain; 21 Red de Investigación SEUP (Sociedad Española de Urgencias Pediátricas) Network, Madrid, Spain; 22 Pediatric Emergency Unit, Hospital Universitario Río Hortega, Valladolid, Spain; 23 University of Switzerland, ., Switzerland; 24 Inselspital, University Hospital of Berne, Berne, Switzerland; 25 Research in European Pediatric Emergency Medicine (REPEM) Network, Leicester, UK; 26 Emergency Department, Tirgu Mures Emergency Clinical County Hospital, Targu Mures, Romania; 27 Alberta Children’s Hospital Research Institute, Calgary, AB Canada; 28 Departments of Pediatrics and Emergency Medicine, University of Calgary, Calgary, AB Canada; 29 Pediatric Emergency Research Canada (PERC) Network, Calgary, Alberta Canada; 30 Division of Pediatric Emergency Medicine, Hospital for Sick Children, Toronto, Canada; 31 SickKids Research Institute, Toronto, Canada; 32 University of Toronto, Toronto, Canada; 33 Division of Emergency Medicine, Children’s Hospital of Philadelphia, Philadelphia, PA USA; 34 Department of Pediatrics, Perelman School of Medicine, University of Pennsylvania, Philadelphia, PA USA; 35 Columbia University Medical Center, New York, USA; 36 Pediatric Emergency Care Applied Research Network (PECARN), New York, USA; 37 Department of Emergency Medicine and Pediatrics, University of Michigan, Ann Arbor, MI USA; 38 Pediatric Care Applied Research Network (PECARN), Utah, USA; 39 University of Utah, Utah, USA; 40 University of New Mexico, Albuquerque, NM USA; 41 Department of Emergency Medicine, Stanford University, Stanford, CA USA; 42 Global Pediatric Emergency Equity Lab at Stanford University, Stanford CA, USA; 43 Pediatric Emergency Medicine Collaborative Research Committee (PEMCRC), Itasca, Illinois USA; 44 Hospital de Pediatría “Prof. Dr. Juan P. Garrahan”, Buenos Aries, Argentina; 45 Universidad de Buenos Aires, Buenos Aries, Argentina; 46 Red de Investigación y Desarrollo de la Emergencia Pediátrica Latinoamericana (RIDEPLA), Leicester, UK; 47 Centro Hospitalario Pereira Rossell de Montevideo, Montevideo, Uruguay; 48 Hospital Nacional de Niños “Dr. Carlos Saenz Herrera”, San José, Costa Rica; 49 Hospital de Santa Maria, Centro Hospitalar Lisboa Norte, Lisbon, Portugal; 50 Laboratório de Farmacologia Clinica e Terapêutica, Faculdade de Medicina, Instituto de Medicina Molecular, Universidade de Lisboa, Lisbon, Portugal; 51 Madras Medical College, Chennai, India; 52 Emergency Medical Services, Western Cape Health, Belville, South Africa; 53 Division of Emergency Medicine, University of Cape Town, Cape Town, South Africa; 54 KK Women’s and Children’s Hospital, Singapore, Singapore; 55 Duke-NUS Medical School, Singapore, Singapore; 56 Emergency Medicine Service, Monash Health, Melbourne, Australia

## Abstract

**Background:**

Acute severe childhood asthma is an infrequent, but potentially life-threatening emergency condition. There is a wide range of different approaches to this condition, with very little supporting evidence, leading to significant variation in practice. To improve knowledge in this area, there must first be consensus on how to conduct clinical trials, so that valid comparisons can be made between future studies. We have formed an international working group comprising paediatricians and emergency physicians from North America, Europe, Asia, the Middle East, Africa, South America, Central America, Australasia and the United Kingdom.

**Methods/design:**

A 5-stage approach will be used: (1) a comprehensive list of outcomes relevant to stakeholders will be compiled through systematic reviews and qualitative interviews with patients, families, and clinicians; (2) Delphi methodology will be applied to reduce the comprehensive list to a core outcome set; (3) we will review current clinical practice guidelines, existing clinical trials, and literature on bedside assessment of asthma severity. We will then identify practice differences in tne clinical assessment of asthma severity, and determine whether further prospective work is needed to achieve agreement on inclusion criteria for clinical trials in acute paediatric asthma in the emergency department (ED) setting; (4) a retrospective chart review in Australia and New Zealand will identify the incidence of serious clinical complications such as intubation, ICU admission, and death in children hospitalized with acute severe asthma. Understanding the incidence of such outcomes will allow us to understand how common (and therefore how feasible) particular outcomes are in asthma in the ED setting; and finally (5) a meeting of the Pediatric Emergency Research Networks (PERN) asthma working group will be held, with invitation of other clinicians interested in acute asthma research, and patients/families. The group will be asked to achieve consensus on a core set of outcomes and to make recommendations for the conduct of clinical trials in acute severe asthma. If this is not possible, the group will agree on a series of prioritized steps to achieve this aim.

**Discussion:**

The development of an international consensus on core outcomes is an important first step towards the development of consensus guidelines and standardised protocols for randomized controlled trials (RCTs) in this population. This will enable us to better interpret and compare future studies, reduce risks of study heterogeneity and outcome reporting bias, and improve the evidence base for the management of this important condition.

## Background

Asthma is a frequent reason for a child to attend the emergency department (ED) [1, 2], and one of the most common reasons for paediatric hospitalization after an ED visit [3]. In the USA, the rate of paediatric ED visits for asthma increased by 13.3% between 2001 and 2010 [4], whilst in the UK it is estimated that a child is admitted to hospital every 20 min due to an asthma attack [5].

Most children with asthma have mild or moderate exacerbations, and respond to first-line treatment with inhaled bronchodilator therapy and systemic steroids [6–9]. However, a proportion of children with severe asthma require more intensive therapies including intravenous (IV) medications, endotracheal intubation and/or admission to intensive care [9–11]. Management of acute severe asthma is complicated by a number of problems, including a large number of treatment options and wide variation in self-reported and actual practice [12].

### Treatment options for acute severe paediatric asthma

Medications used for acute severe paediatric asthma include IV bronchodilators (such as salbutamol/albuterol [13], terbutaline [14], magnesium [15], or aminophylline [16]), nebulized magnesium [17], inhaled heliox [18], and IV ketamine [19]. Respiratory support with non-invasive and/or invasive ventilation may also be used if there is a poor response to intensive medical treatment [20].

A Cochrane review of the addition of IV beta_2_-agonists to inhaled beta_2_-agonists concluded that until further high-quality, adequately powered trials are conducted, “it is not possible to form a robust evaluation of the addition of IV beta_2_-agonists in … severe acute asthma” [13].

Intravenous magnesium sulphate (MgSO_4_) appears to be safe and beneficial in severe asthma in adults [21]. Data on the use of MgSO_4_ in children are not as robust, but appear promising [15]. Inhaled MgSO_4_ is also used in acute severe asthma [22], although recent research has not confirmed a clinical benefit, rather that “no harm is done” and that it “may be helpful” in some patients [17].

When added to inhaled bronchodilators and systemic corticosteroids, IV aminophylline may lead to an earlier improvement in lung function, but may be associated with a significant increase in the rate of vomiting [23]. There is insufficient evidence for any effect of aminophylline infusion on other useful clinical outcomes such as reducing the intubation rate, frequency of intensive care admission, or hospital length of stay [24, 25]. Cochrane reviews of heliox [18] and ketamine [26] as therapies for severe asthma have not demonstrated any consistent benefit.

### Variation in practice

Given the lack of useful comparative studies, variation in the management of acute severe asthma in children is considerable. A recent survey in the UK and Ireland found that over half of front-line emergency and general paediatricians preferred salbutamol as first-line IV treatment, whilst 28% preferred MgSO_4_, and 15% preferred aminophylline [12]. An earlier survey of paediatric emergency physicians in Australia and New Zealand found that aminophylline was used by 45%, IV MgSO_4_ by 55%, and IV salbutamol by 87% of respondents [27]. Whilst magnesium and salbutamol are listed as second-line and third-line bronchodilators for management of acute severe paediatric asthma in all Australian guidelines [28], only some guidelines include aminophylline [29]. A recent prospective study in 24 EDs in the UK and Ireland found wide variation in the use of IV bronchodilator treatment for acute paediatric asthma ranging from 0% to 19.4% [11].

The United States National Heart, Lung and Blood Institute (NHLBI) Expert Panel Report guidelines suggest IV MgSO_4_ or inhaled heliox for impending respiratory failure or persistently severe asthma. The guidelines state there are insufficient data to make recommendations on intravenous beta_2_-agonists and IV leukotriene receptor antagonists, and specifically recommend against aminophylline [30].

British guidelines recommend considering IV MgS0_4_, IV aminophylline, or IV salbutamol for a child with severe or life-threatening asthma unresponsive to first-line treatment, with a preference for use of MgS0_4_ as the first option [31].

The Global Initiative for Asthma (GINA) guidelines for acute severe asthma recommend against IV aminophylline. The guidelines suggest that IV MgSO_4_ can be considered for acute severe asthma, and that IV terbutaline may be given to a severely ill child where inhaled therapy is not possible, although there is “no evidence to support the routine use of IV beta_2_-agonists in patients with severe asthma exacerbations” [32]. Thus, there is considerable geographic variation in clinician preference, guidelines, and actual use of IV agents for acute severe paediatric asthma, reflecting the overall paucity of high-quality evidence to inform clinicians [16].

### Consistent outcome measures are lacking

Previous reviews of acute asthma have not been successful in making meaningful comparisons between treatments, due to the varying outcome measures used in many trials [23, 25]. Inconsistent selection, measurement, and reporting of outcomes has been noted both in selecting domains and measurement instruments. These issues could be addressed with the development and application of agreed standardized sets of outcomes, i.e. “core outcome sets”, that are important to relevant stakeholders [33]. These could include subjective measures relevant to patients, economic outcomes, and physiologic outcomes (such as asthma severity scores) [22]. Review authors have suggested the need to focus on clinically important outcomes such as frequency of admission to hospital and the intensive care unit (ICU), length of hospital stay, and relapse rates [13].

Acute asthma has been highlighted as a research priority by multiple paediatric emergency medicine research networks [34–36]. However, meaningful progress is unlikely until there is consensus on a set of core outcome measures that are relevant to clinicians, patients, families, and healthcare funders.

### Context – Pediatric Emergency Research Networks (PERN)

Over the last 10 years, there has been significant worldwide collaboration between major Pediatric Emergency Research Networks. The Pediatric Emergency Research Networks (PERN) collaboration involves research organisations from USA, Canada, Europe, Spain, the UK and Ireland, Australia and New Zealand, and South and Central America, as well as contributions from additional countries outside a formal research network [37]. The collaboration has enabled a number of successful international multicentre projects to be completed [38–43].

## Aims and objectives

### Aims

The PERN asthma working group was formed in 2017, with the aims of developing consensus evidence-based acute asthma outcome measures (with the input of patients, families, and clinicians), and international consensus guidelines for the conduct and reporting of clinical trials of therapies for acute asthma exacerbations in children attending emergency departments. We aim for our core outcome set to be applicable to children with severe acute asthma receiving any therapeutic intervention, including inhaled therapies, parenteral treatment, or oxygen/ventilatory therapy. Currently, the working group comprises emergency physicians and paediatricians from seventeen countries. This paper describes the proposed methodology for our work (current as of July 2019), which has been registered with the Core Outcome Measures in Effectiveness Trials (COMET) initiative [44].

## Methods/design

### Overview

**Fig. 1:**
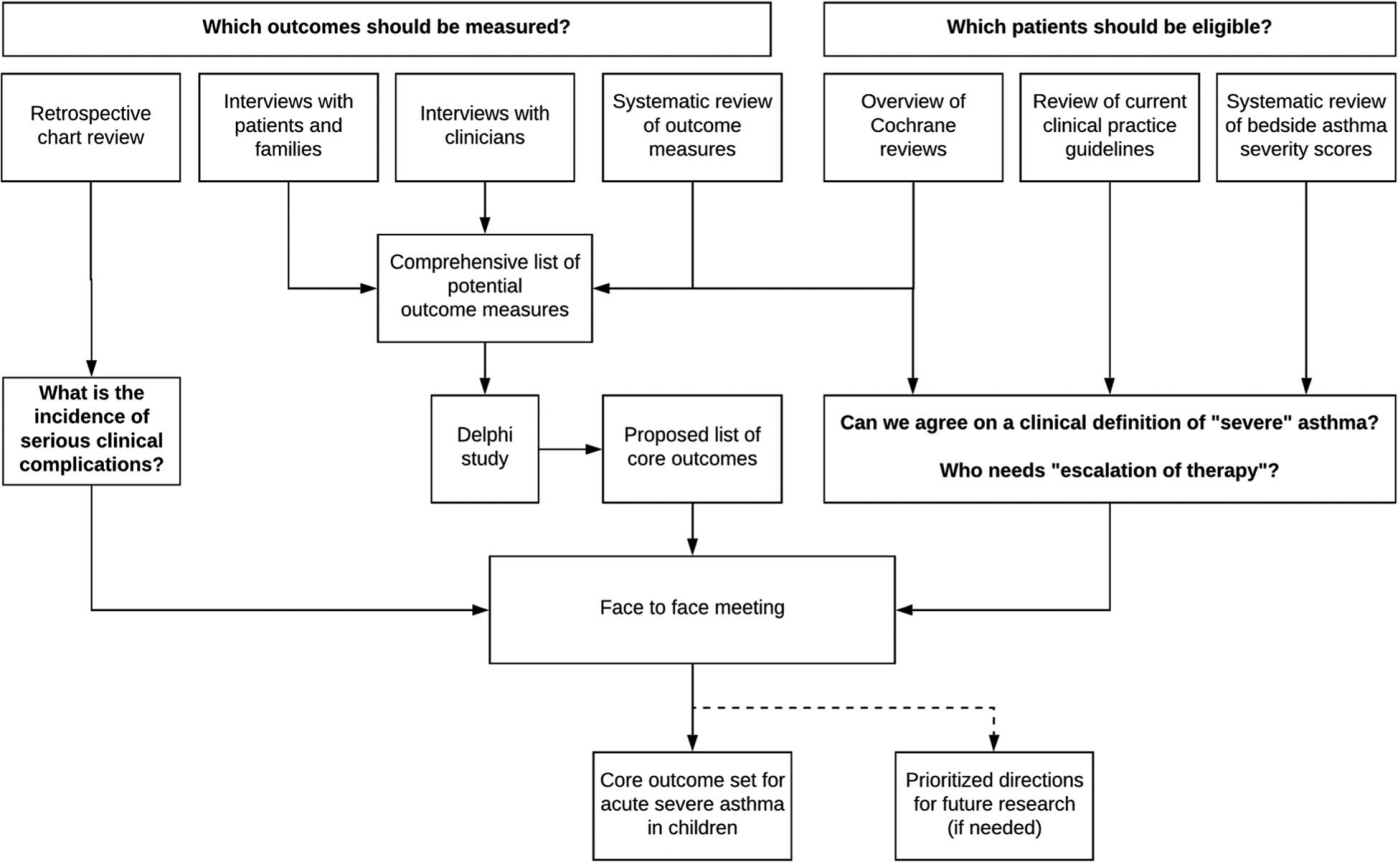
Overview of methodology to develop core outcome set in acute severe paediatric asthma

Core outcome set development will follow recent guidance from the COMET initiative [45]. A five-stage approach will be used (Fig. 1). A comprehensive list of outcomes relevant to stakeholders will be compiled through a systematic review of outcomes reported in existing trials, supplemented by qualitative interview studies with patients, families, and clinicians. Second, the comprehensive list will be reduced to a core outcome set using Delphi methodology involving patients, families, and clinicians.

Third, to inform consensus discussions on inclusion criteria and outcome selection for future RCTs in acute severe paediatric asthma in the ED setting, we will review current clinical practice guidelines, existing clinical trials, and literature on bedside asthma severity scores. We will then identify practice differences in bedside assessment of asthma severity, and determine whether further prospective work is needed.

Fourth, a retrospective chart review in Australia and New Zealand will enable us to identify the incidence of serious clinical complications such as intubation, ICU admission, and death in children hospitalized with acute severe asthma. Understanding the incidence of such outcomes will allow us to understand how common (and therefore how feasible) particular outcomes are in asthma in the ED setting.

Finally, a meeting of the PERN asthma working group will be held, with invitation of other clinicians interested in acute asthma research, and patients/families. Results of the initial steps outlined above will be presented, and the group will be asked to achieve consensus on a core set of outcomes and to make recommendations for the conduct of clinical trials on acute severe asthma. If this is not possible, the group will agree on a series of prioritized steps to achieve this aim.

### Project 1: developing a comprehensive list of outcome measures

In order to develop a comprehensive list of outcome measures, we have performed a systematic review of outcomes reported in existing trials, which will be complemented by an overview of Cochrane Reviews, and qualitative interview studies with patients, families, and clinicians.

#### Systematic review

We have recently conducted a systematic review of primary and secondary outcomes used in trials of IV bronchodilators in children with acute severe asthma. We searched MEDLINE, EMBASE, Cochrane CENTRAL, and the World Health Organization International Clinical Trials Registry Platform for randomized trials in children (younger than 18 years) with acute severe asthma, comparing IV bronchodilator therapy to another treatment.

Data extraction included authors, year of publication, geographic location, number of patients, primary, secondary, and other outcomes used, and study interventions including dosing and timing of medication administered. The systematic review was prospectively registered in the PROSPERO database (CRD42017055331), and has recently been published [46]. We identified 35 published papers and four registered study protocols. We identified 56 primary outcomes, the most common being a clinical asthma score (23/56; 41%). Other primary outcomes identified included bedside tests of respiratory function (11/56; 20%) and measures of length of stay (9/56; 16%). There were a total of 60 different secondary outcomes; the most common were various measures of length of stay (24/60; 40%) and adverse events (11/60; 18%) [46].

#### Overview of Cochrane Reviews

Members of the PERN asthma working group are also undertaking an overview of Cochrane Reviews to summarize Cochrane Reviews on the efficacy and safety of interventions for escalation of therapy for acute exacerbations of asthma in children. The protocol for this overview has been published [47]. Additional objectives of the overview are to (1) identify gaps in the current evidence base that will inform recommendations for future research and subsequent Cochrane Reviews; and (2) to categorize information on reported outcome measures used in trials of escalation of treatment for acute exacerbations of asthma in children.

#### Qualitative interview studies: patients/families and clinicians

This part of the project aims to assess the perspectives of patients, families, and clinicians regarding their experience of acute severe paediatric asthma. Qualitative methods are recommended to identify outcomes important to stakeholders, help understand why these outcomes are important, and identify appropriate language to use when presenting these outcomes in later surveys [48]. These perspectives will be combined with a similar study of clinicians, and the results of the systematic review to develop a comprehensive list of outcome measures. This list will directly inform the development of a planned Delphi survey (see below).

To ensure global representation and maximum diversity, we aim to analyse the perspectives from a number of patient and clinician groups from multiple hospitals in a variety of settings around the world. Patients and families will be purposively sample d[45], with selection of children with recent experience of acute severe asthma exacerbations requiring attendance in an emergency department and admission to hospital, managed in a variety of hospitals in a variety of healthcare systems. We aim to include clinician groups from multiple EDs, paediatric wards, and ICUs. Initially, we will select clinicians from the already established asthma working group, and then - if further participants are required - expand to include intensive care physicians and hospitalists/paediatricians.

The specific objectives of the patient/family interviews are to (1) determine which aspects of clinical care and which outcomes are most important to patients and families in this setting; (2) determine the underlying reasons why these outcomes are important; and (3) identify possible participants for a patient steering group and participation in a consensus meeting. For the clinician interviews, our first two objectives are the same as for the patient/family interviews, whilst a third objective is to determine how clinicians define acute severe exacerbation of asthma.

Ethics approval for the interview studies has been obtained in Australia; additional local ethics approval will be sought where relevant. An overview of the methods of the interview studies for clinicians and patients/families is presented in Table 1.

**Table 1:** Methodology for qualitative interview studies

	Patient/family interview study	Clinician interview study
Hospital selection	• Ability to conduct the patient interviews • Overall diversity (geographic, health system, and socio-demographic differences) of hospitals selected • Maximum of 4 sites chosen from any one research network • No more than 3 hospitals from the same country	• Emails inviting physicians to participate will be distributed to the existing Pediatric Emergency Research Network (PERN) asthma working group • Aim to include both tertiary paediatric centres and mixed hospitals
Participant selection	• Each hospital to arrange interviews with the family members of 2 patients. The patient will also be able to participate if deemed mature enough by their parent/carer • Eligibility: admitted to the hospital via the emergency department (ED) with an acute exacerbation of asthma • Family to be approached while the child is still an inpatient on the hospital ward, once the child is stable	• Working group members will be eligible to be interviewed • Group members will also be asked to approach a local colleague from another medical discipline (e.g. emergency physician, paediatrician, intensivist or respiratory paediatrician) to seek their participation • Maximum of 5 clinicians from any single country • No more than 2 clinicians from the same hospital
Interview format	• Semi-structured face-to-face interviews by local investigators at each site • Interview will take place at the patient’s bedside, or in an interview room within the ward setting • Audio recorded and transcribed	• Semi-structured telephone interview by the central study investigators (CG and JMcC), based in Adelaide, Australia • Interviews will be conducted in English, using telephone or Internet-based telecommunication (e.g. Skype) • Audio recorded and transcribed
Thematic analysis taxonomy	• Outcomes in medical research, comprising mortality/survival, physiological/clinical, life impact, resource use, and adverse events/effects [42]	• Outcomes in medical research, comprising mortality/survival, physiological/clinical, life impact, resource use, and adverse events/effects [42] • Theoretical domains framework [43]
Review of patient cohort and determination of thematic saturation	• After re-coding and analysis of 2 interviews from 5 participating sites (a total of 10 interviews), the project steering group will review the content of the themes obtained, and assess the demographic and clinical characteristics of participating families • Aim to balance between patient age, severity of asthma exacerbation, and whether or not the patient has had previous exacerbations • Determine whether further interviews need to be conducted in a particular population. For example, the steering group may recommend more recruitment of pre-schoolers vs school-aged children, those with severe/life-threatening asthma vs those with milder disease, those from low-to-middle income countries vs high-income countries, or those with previous asthma exacerbations vs those with a first episode • Interview schedule will be reviewed to determine if any changes are required	• After re-coding and analysis of interviews from 5 participating sites (a total of 10 interviews), the project steering group will review the content of the themes obtained, and assess the demographic characteristics of participating clinicians • Aim to balance between type of health system (high-income country vs low-to-middle income country), and type of practitioner (emergency specialist vs paediatrician vs intensivist) • Determine whether further interviews need to be conducted in a particular population. For example, the steering group may recommend more recruitment of paediatricians or intensivists, or more recruitment from high-income countries • Interview schedule will be reviewed to determine if any changes are required
Reassessment and determination of thematic saturation	• Reassessment every 5 interviews after the initial 10 until it is determined that thematic saturation is reached and that a representative sample of all important patient types has been achieved • Thematic saturation reached when 3 consecutive interviews have been completed with no new themes emerging	

Invitations to participate will be distributed via email through the partner networks that contribute to PERN, and to clinicians associated with the PERN asthma working group. Copies of the interview schedules are available as Additional files 1 and 2.

To provide a clinical context to each patient interview, there will be focused review of the relevant medical records for included patients. We will extract details of the patient’s age, gender, previous asthma history, and treatment administered during the index hospitalisation.

### Data analysis

Any identifying data will be removed from the interview transcript prior to analysis. Patient family interviews conducted in a language other than English will be translated into English for the purpose of analysis. The translation will be checked by the original interviewer.

Thematic analysis will be performed independently by the principal investigator for each study (SC for the patient/family interview study and CG for the clinician interview study) and one other investigator, based upon the taxonomy developed for outcomes in medical research, comprising mortality/survival, physiological/clinical, life impact, resource use, and adverse events/effects [49]. Additional taxonomy based on the Theoretical Domains Framework (TDF) [50] will be used for the clinician interviews. The themes and coding will then be reviewed and cross-checked by the original interviewer. N-vivo qualitative analytic software (QSR International, Melbourne, Australia) will be used for analysis.

### Sample size

Due to the qualitative nature of the studies, we have not pre-determined a sample size. Table 1 provides details on how thematic saturation will be determined for each study. We are aiming to obtain representative views from diverse populations around the world, so anticipate the involvement of at least ten hospitals for each study.

### Data management and storage

De-identified transcripts will be collected and stored in a password-protected file at Monash Health, Melbourne, Australia (patient interview study) and at Women and Children’s Hospital, Adelaide (clinician interview study). As per the National Health & Medical Research Council, we will keep original data for at least 7 years.

### Ethical aspects

The patient interview study has been approved by the Monash Health Human Research Ethics Committee (RES-18-0000-530A), whilst the clinician interview study has been approved by the Women’s and Children’s Health Network Human Research Ethics Committee (HREC/18/WCHN/120). Site-specific approval will be obtained at each site prior to commencement of local data collection.

### Project 2: prioritising outcome measures: Delphi process

With information from systematic reviews and qualitative interviews, we will develop a comprehensive list of outcome measures. We will then conduct a prioritization exercise using modified Delphi methodology.

We intend to have two independent panels for the Delphi study: a patient/family panel and a clinician/researcher panel. Those participating in the qualitative research projects will be invited to participate in the Delphi study. Similar patient/family participants will be recruited from hospitals involved in the PERN member networks, and third-party asthma organisations, such as the National Asthma Council Australia, Asthma UK, Asthma and Allergy Foundation of America, and Asthma Canada. Additional clinician/researcher participants will be recruited through the PERN member networks and circulation of an invitation email by members of the PERN asthma working group to their local and national paediatric and/or emergency medicine organisations. Social media will also be used to recruit participants.

Due to the importance of minimising attrition during the Delphi process [45], the requirement for participants to complete all rounds will be emphasized during recruitment, and reinforced during the informed consent process and ongoing communications (for example, using email reminders) once the study has commenced. The list of outcomes will be presented in plain, non-technical language. We intend to use an online survey platform such as Delphi Manager (http://cometinitiative.org/DelphiManager/) to conduct the study, and plan to create and circulate simultaneous surveys in English, Spanish, and potentially other languages to allow participation of patients, families, and clinicians based in the various countries contributing to the project. Reminders will be sent to non-responders at weekly intervals for 2 weeks for each round of the Delphi study. All questions and free-text responses will be professionally translated. Prior to commencement, we will seek approval from relevant human research ethics committees.

#### Delphi round 1

In the first round, participants will be registered on line, and provide basic demographic information. Clinicians/researchers will be asked for details of their place of work and role (emergency physician, paediatrician, intensivist, etc.), whilst patients/family members will be asked for details on their most recent hospital experience, and number of hospitalizations and ICU admissions. To enable identification of individuals completing each round, each participant will be allocated a unique identifier.

The comprehensive list of outcomes will be presented to participants in plain language. Questions will be grouped into topics (e.g. vital signs, length of stay measures, etc.), and general questions will precede specific ones. Participants will be asked to rate the importance of each of the outcomes on a 9-point Likert scale, with 1 labeled as “not important”, and 9 labeled as “critical”. There will be space for participants to provide optional free-text comments for each choice. There will also be a free-text box at the end of the survey for respondents to suggest any additional outcomes not included in the questionnaire.

#### Delphi round 2

Responses to the first Delphi round will be analyzed separately by the stakeholder group. No items will be removed. Open-ended responses will be collated, translated into English as necessary, and those suggested by two or more respondents will be added to the initial list.

The second round of the Delphi survey will present all items from round 1 as well as new open-ended responses. Items that were ranked in round 1 will be presented alongside feedback to participants with presentation of scores from both the patient/family group and the clinician/researcher group.

#### Delphi round 3

Items for round 3 will be based upon the results of the second round, with inclusion if they are rated 7–9 (on the 9-point Likert scale) by 50% or more participants and 1–3 by no more than 15% of participants in at least one stakeholder group [45]. Each item will be presented alongside round 2 scores from both stakeholder groups.

The survey for round 3 will also allow respondents to indicate their willingness to be contacted for a planned face-to-face meeting within the following 6–12 months to discuss the results of the study and determine a final list of outcome measures.

During all survey rounds, the questionnaires will be designed so that participants are not able to skip any questions or leave any questions blank, to ensure there are no missing data.

#### Definition of consensus

The core set of outcomes from the Delphi survey will be selected after the third round of the survey according to the following criteria: (1) 70% of participants scoring outcomes as 7–9 and 15% or less scoring 1–3 by both stakeholder groups; or (2) 90% or more scoring 7–9 from either stakeholder group [45].

### Project 3: reviewing current assessment tools for bedside assessment of asthma severity

To understand current assessment practices, a review of currently used clinical guidelines and inclusion criteria for existing clinical trials is ongoing, along with a review of the literature on bedside asthma severity scores. The primary objective is to describe and compare acute severity assessment recommendations on childhood asthma exacerbations within and between geographic regions. This will be critical to ensure that patients enrolled in future studies of asthma treatments are comparable across regions. Secondary objectives are to compare management recommendations, and to assess the quality of existing guidelines, in order to clarify the extent to which current practice reflects the available evidence, and highlight areas for further research.

#### Sampling of clinical practice guidelines

We will sample clinical practice guidelines by distributing emails inviting physicians and hospitals to participate via the eight partner networks that contribute to PERN, and to the clinicians associated with the PERN asthma working group. The email recipients will be requested to forward the email to other physicians and hospitals within their local geographic region and/or research network. The request for participation will also be shared on social media. This *snowball* approach, building on existing formal and informal professional and academic relationships, will allow sampling within countries without formal organised research networks.

Each participating hospital will be asked to provide copies and/or web links to their current clinical asthma guideline, and any regional or national clinical guideline. Documents may be entitled clinical protocols, clinical guidelines, care pathways, or other similar titles. However, for the purposes of this study, any document that provides recommendations on severity assessment and treatment for children > 1 year of age presenting to the ED/hospital with acute onset of wheezing or asthma will be considered eligible for inclusion.

The clinical guideline project has been deemed a quality assurance activity by the Monash Health Human Research Ethics Committee (RES-18-0000-525Q), and had obtained details of more than 110 local, national, and international asthma guidelines by July 2019.

### Data abstraction: guideline content

Components of each clinical guideline will be independently abstracted by two reviewers. Abstracted data will be recorded on a purpose-designed password-protected spreadsheet. Any discrepancies in data abstraction between the two reviewers will be discussed. If discrepancies remain, a third reviewer will be consulted, and a decision will be made by consensus.

Guidelines that are written in languages other than English will be abstracted by two investigators who are fluent in English and in the language in which they are written.

Specific data will be abstracted on:

1. Definition of asthma (including age range)
2. Assessment of acute asthma severity, including use of asthma severity scores. This will include abstraction of recommendations for when to utilise these scores (i.e. at triage, within the ED, while an inpatient), and whether the scores include clinical findings only, or additional investigations.
3. Recommendations and thresholds for initiating treatments, including:
  - Inhaled bronchodilator therapy;
  - Enteral, parenteral, or inhaled corticosteroids;
  - Adjunctive inhaled therapy;
  - IV/subcutaneous/intramuscular bronchodilator medications;
  - Oxygen therapy (including devices and flow rates);
  - Non-invasive or invasive ventilation;
  - Admission/discharge;
  - ICU admission/interhospital transfer;
  - Treatment at discharge;
  - Follow-up management.
4. Specific guidance for asthma complications (such as pneumothorax, atelectasis).
5. Each recommendation will be abstracted in terms of direction (to use/not use), and, if available, strength of recommendation and quality of supporting evidence. Thresholds for treatment initiation will be abstracted according to history, examination, bedside tests of lung function, clinical asthma scores, and laboratory tests.

A copy of the data abstraction sheet is provided in the Additional file 3.

Comparisons will be made within each geographic region/research network, and across all guidelines. Specific comparisons will be made on treatment recommendations for severe asthma within each geographic region, including inhaled magnesium, IV beta-agonists, IV aminophylline, IV magnesium, and non-invasive or invasive ventilation. Agreement between recommendations in the guidelines will be assessed.

### Data analysis: guideline quality

Each clinical practice guideline will be assessed using the AGREE-II instrument [51]. Each guideline will be independently assessed by at least two appraisers to increase the reliability of the instrument [51]. All raters will undergo specific training on the use of the AGREE-II instrument. Guideline quality will be assessed overall and for each of the six domains examined by the AGREE-II instrument. Comparisons will be made between regions/research networks and all other guidelines, and between guidelines published prior to 2010 and those from 2010 and onwards.

### Data management and storage

Data will be collected using a dedicated study email address, and extracted data stored in a password-protected electronic folder at Monash Health. Data will be stored for 7 years after the completion of the study.

#### Literature search and systematic review for asthma severity scores

We will perform a systematic review to determine the reliability and validity of clinical bedside scores used for assessment of asthma and/or wheeze in children. Search terms will include “[asthma OR wheeze] AND [validity OR validation OR reliability] AND [score OR scale OR assessment OR index]”, with appropriate modifications for each database, and be limited to children aged 0–18 years. We will also search for each clinical severity score identified by sampling of clinical practice guidelines. The search will be conducted in the Pubmed, Embase, Cochrane library, National Guideline Clearinghouse, and CINAHL databases. Each scoring system will be assessed with quality criteria relating to utility, reliability, and validity [52].

### Step 4: clinical outcome prevalence in acute severe paediatric asthma

We will conduct a multi-centre, retrospective cohort study of eligible children diagnosed with asthma or wheeze in 19 Australasian EDs associated with the Paediatric Research in Emergency Departments International Collaborative (PREDICT) network.

The study population will be children aged 1–17 years (up to but not including 18th birthday) presenting to a participating ED with a discharge diagnosis of asthma/wheeze, who are administered inhaled or IV medication or receive invasive or non-invasive respiratory support. We will exclude children with a final ED diagnosis of bronchiolitis and no bronchodilator administered, those with a diagnosis of foreign body inhalation, and those receiving IV bronchodilator treatment before arrival.

Each site investigator will identify a list of medical records of all children meeting inclusion criteria presenting between 1 November 2015 and 31 October 2016. These records will be identified using the local ED information system and hospital medical records, using the following ICD-10 codes: “Asthma” or “Childhood asthma” (ICD-10 code: J45, J46); “Wheezing” (ICD-10 code: R06.2); and “Acute bronchiolitis” (ICD-10 code: J21).

Each child attending the ED with asthma/wheeze is eligible for inclusion, even if the child has previously been included in the study. Each record will be assigned consecutive numbers by the medical records department, and then be screened for eligibility. Required information will be obtained from charts by trained abstractors.

The main outcome measure is the prevalence of IV bronchodilator therapy administration. Secondary outcome measures include type of bronchodilator therapy used; non-invasive respiratory support (continuous positive airway pressure (CPAP)/bi-level positive airway pressure (BiPap), and high-flow nasal oxygen therapy); prevalence and duration of intubation and mechanical ventilation; rates of hospital admission, ICU admission, and interhospital transfers; complication rates for pneumothorax, hypokalaemia requiring replacement therapy, hypotension requiring an IV fluid bolus of at least 10 mL/kg, and arrhythmias requiring electrocardiogram (ECG) monitoring or antiarrhythmic therapy.

### Data storage and analysis

De-identified data will entered into a protected electronic database at each participating hospital using Research Electronic Data Capture (REDCap) [53], and be housed at a central data processing site at Murdoch Children’s Research Institute (MCRI), Parkville, Victoria. All study investigators and the biostatistician will have full access to the final study dataset. The complete data set will be analysed at Monash Children’s Hospital, Clayton, Victoria.

Data will be descriptively analysed. Binary outcomes will be presented as proportions with 95% confidence intervals. For continuous outcomes, data will be presented as means and standard deviations (normally distributed data) or medians and interquartile ranges (non-parametric data).

For categorical data, comparisons will be made with calculation of odds ratio, and significance determined using chi-square analysis. For non-parametric continuous data, comparisons will be made using the Mann-Whitney U test (two-group comparisons) and the Kruskall-Wallis test (three-group comparisons). For normally distributed continuous data, two-group comparisons will be made using the independent samples *t* test, whilst three-group comparisons will be made using analysis of variance (ANOVA).

### Exploratory modeling analysis

Multiple logistic regression will be used to assess predictors of ICU admission and other binary outcomes, whilst multiple linear regression will be used to assess continuous outcomes such as hospital length of stay.

We will use generalized linear mixed effects models to account for multiple admissions and clustering of patients within hospitals. Hospitals will be included as random effects in these models and the clustered nature of the data will be modelled by fitting the appropriate covariance structure. Analysis will be performed using the PROC GLIMMIX procedure available in SAS software.

### Ethical aspects

The project has been approved by the Monash Health Human Research Ethics Committee (RES-17-0000-238 L), and site-specific approval has been obtained at each site prior to commencement of data collection.

### Sample size

The rate of administration of IV therapy for children in Australia and New Zealand is currently unknown. Based on the approximately 3% rate of IV therapy in the UK study [11], we anticipate the need to review approximately 1000 eligible charts at each site to obtain 30–40 cases in which IV bronchodilators are administered. Data collection at 19 hospitals will provide data on approximately 570 children with asthma requiring IV therapy. Each site will be requested to collect data on 1000 patients or 12 months of data (whichever is less).

If more than 1000 eligible medical records are identified on the initial screen of medical records, a random number generator will be used to create a series of random numbers corresponding to the medical records. Screening will then occur in order of the generated random numbers until 1000 eligible records are identified.

This project will provide information on the incidence of severe paediatric asthma/wheeze in Australia and New Zealand; prevalence of complications and clinical outcomes; and patterns of IV and other escalated therapy for these children. This information will inform discusions relating to the feasibility of specific clinical outcome measures to power trials in children with severe asthma exacerbations. For example, if a particular clinical outcome (e.g. intubation, pneumothorax) was found to be very rare, it would not be feasible to power a randomized controlled study to detect a clinically meaningful difference between treatment groups for that outcome.

### Step 5: consensus meeting and next steps

Once results from steps 2, 3, and 4 are available, a half day face-to-face meeting will be arranged in Melbourne, Australia, in which international investigators can participate by teleconference. Invitations will be sent to all study steering group members, Delphi study participants who have indicated a willingness to be involved in such a meeting, and representatives of major stakeholders such as general practitioners, patient advocacy groups, and specialist medical and nursing colleges.

The meeting will enable discussion of the results of the Delphi survey to agree a final set of core outcomes, and undertake additional voting if required. Attendees will be sent a reminder of their personal Delphi scoring prior to the meeting. We will seek to ratify outcomes that have met the predetermined definition for the Delphi survey, and to further discuss and then make a decision upon those outcomes that had not met the consensus threshold prior to the meeting. Additional discussion will then occur on how best to measure the chosen outcomes, and identification of any further research required to better inform decisions about measurement.

Once the meeting has been completed, we intend to produce a consensus statement relating to core outcome measures in acute severe asthma. This will be drafted and a copy circulated to all those involved in the project for comment prior to finalization and distribution.

## Trial status

At the time of submission of this paper (August 2019), the trial status is as follows:

- Systematic review of outcome measures: completed and published in November 2018.
- Overview of Cochrane Reviews: protocol completed and published March 2018. Literature search and data collection complete. Manuscript preparation ongoing.
- Patient interviews (protocol version 1.4, date: 17 September 2018). Data collection commenced February 2019. Anticipated completion mid-2020.
- Clinician interviews (protocol version 1.3, date: 31 August 2018). Data collection commenced May 2019. Anticipated completion mid-2020.
- Review of clinical practice guidleines (protocol version 1.3, date: 3 August 2018). Data collection commenced September 2018, data extraction ongoing. Anticipated completion mid-2020.
- Retrospective cohort study (current protocol version 2.8, date: 12 December 2018 - amended due to additional participating hospitals; original approved protocol version 2.3, date 30 May 2017). Data collection commenced June 2017, data collection ongoing. Anticipated completion late 2020.
- Yet to commence: Delphi study, review of asthma severity scores, consensus meeting.

## Discussion

There is currently no published, globally applicable, core outcome set for acute severe paediatric asthma. Studies comparing IV treatment modalities exhibit great variation in the type, number, and timing of outcome measures used.

The development of an international consensus on core outcomes is an important first step towards the development of consensus guidelines and standardised protocols for RCTs in this population. This will enable us to better interpret and compare future studies, reduce risks of study heterogeneity and outcome reporting bias, and improve the evidence base for the management of this important condition.

Once developed, we aim to disseminate our core outcome set and consensus guidelines widely through presentation at relevant scientific meetings and publication in relevant journals. We will adhere to guidelines for authorship outlined by the International Committee of Medical Journal Editors [54]. We will also enable study participants to register an email address for communication of study results.

## Supplementary information

**Additional file 1.** Interview schedule.

**Additional file 2.** Interview schedule.

**Additional file 3.** Data extraction sheet.

**Additional file 4.** SPIRIT 2013 Checklist: Recommended items to address in a clinical trial protocol and related documents.

## Data Availability

Not applicable.
