# Supplementary material for: Acute severe paediatric asthma: study protocol for the development of a core outcome set, a Pediatric Emergency Reserarch Networks (PERN) study
Source: Trials. 2020 Jan 13;21:72. doi: 10.1186/s13063-019-3785-6 (PMC6956506; doi:10.1186/s13063-019-3785-6)
Supplement: Supplementary file 1 — Additional file 1. Interview schedule. [file 13063_2019_3785_MOESM1_ESM.docx]

**Additional file 1: Interview schedule**

**Draft interview schedule**

- Introduction. Note that interview will be recorded, and a transcript provided for checking.

*I understand that you treat children who present/are admitted to hospital with exacerbation of their asthma. As you are aware, most children improve with systemic corticosteroids and inhaled beta-agonists. Some children do not respond to this treatment, and require escalation in treatment, such as inhaled magnesium, parenteral bronchodilators, or respiratory support. I would like to discuss with you today how you assess and treat these children, including how you would define a severe exacerbation of asthma. Do you give your verbal consent to be involved in this study and agree that I will document this verbal consent on the CRF sheet?*

- - How do you decide a child you are seeing in the emergency department that you work has asthma? And what criteria do you use to differentiate severity? (observations or clinical signs)
  - Can you describe the measures/signs that you use to decide a child should be classified as experiencing a **severe** exacerbation of asthma?
    - Do you routinely use an asthma clinical score to assist with this definition and if so, which one?
  - What factors do you use to decide if the child warrants use of:
    - Inhaled magnesium?
    - Intravenous bronchodilators?
    - High-flow nasal oxygen?
    - Non-invasive ventilation?
  - Does access to resources influence the treatment you use?
  - Do you feel you have the necessary skills to administer the treatment regime you think should be used?
  - With more invasive treatment (such as intravenous bronchodilators, non-invasive ventilation, etc) what parameters or clinical symptoms/signs are you trying to change?
  - How do you measure this?
  - What worries you most about children with acute severe asthma?
  - How do you decide which treatment regime to use?
    - *PROMPTS*
      - *Local guidelines? (What do you think your local guidelines are based on?)*
      - *Consensus amongst colleagues in your department?*
      - *Personal experience?*
      - *Knowledge of current research data?*
  - Have you changed your practice in the last 1-2 years? In the last 5 years?
    - *If yes, what was this change based on?*
  - Do you feel you use “the best” treatment regime?
    - *Why do you think this is the best treatment regime?*
    - *How similar do you think your treatment is to that given by your colleagues?*
    - *In the same department? In the same hospital? Internationally?*
    - *If there are differences – why do these differences exist?*
  - How do you define treatment success?
  - If we were doing research looking at how well a treatment worked in children with acute/severe asthma what outcomes would you think are important?
    - *Why are these outcomes important?*
    - *What information do you need or questions do you need answering from research to help you manage this condition?*
    - *What evidence gaps do you think there are?*
- Conclude interview. Check for any missing concepts, anything else the interviewee would like to say.
- Questions regarding involvement in later follow-up work.
- Thank participant.
