# Supplementary material for: Acute severe paediatric asthma: study protocol for the development of a core outcome set, a Pediatric Emergency Reserarch Networks (PERN) study
Source: Trials. 2020 Jan 13;21:72. doi: 10.1186/s13063-019-3785-6 (PMC6956506; doi:10.1186/s13063-019-3785-6)
Supplement: Supplementary file 2 — Additional file 2. Interview schedule. [file 13063_2019_3785_MOESM2_ESM.docx]

**Additional file 2: Interview schedule**

- Introduction. Note that interview will be recorded, and a transcript provided for checking.
- I understand that XXX (child) was admitted to hospital with asthma. Tell me about your experience of XXX being in hospital?
  - What were the things that were most important to you?
    - During XXX’s time in the emergency department?
    - Ward?
    - ICU?
    - Ambulance?
    - During interhospital transfer?
    - Upon discharge home?
  - Was there anything else that worried you, or that you were concerned about? Were these issues addressed during your time in hospital? If not, why not?
  - Is there anything that should have been done differently?
  - What do you think would be important to other parents / families if their child was in a similar situation to XXX’s? Why are these things important?
  - What if their child was really ill and going to an intensive care unit?
  - What do you think would make your experience different to others?
- Before this visit, have you had to go to hospital before with asthma?
  - How different was this experience?
  - What do you know now that you didn’t know then?
  - How could the other visit(s) have been improved?
- If we were doing research looking at a new or different treatment for children with a bad attack of asthma, what do you think are the most important things for us to look at?
  - If we were going to introduce a new treatment, how do you think we could tell it was doing a good job? *[Explanation (if needed) – examples of process outcomes (time to treatment, communication), clinical outcomes (change in symptoms) and psychological outcomes (reassurance)].* Why are these things important?
- Conclude interview. Check for any missing concepts, anything else the interviewee would like to say.
- Questions regarding involvement in later follow-up work.
- Thank participant.

Date of interview __ / __ / ____

Setting: Ward Intensive care Outpatients

Language interview conducted in: _______________________

Who was interviewed: Mother Father

Sibling Child

Uncle Aunt

Other (specify) __________________
