# Supplementary material for: Acute severe paediatric asthma: study protocol for the development of a core outcome set, a Pediatric Emergency Reserarch Networks (PERN) study
Source: Trials. 2020 Jan 13;21:72. doi: 10.1186/s13063-019-3785-6 (PMC6956506; doi:10.1186/s13063-019-3785-6)
Supplement: Supplementary file 3 — Additional file 3. Data extraction sheet. [file 13063_2019_3785_MOESM3_ESM.docx]

## Additional file 3: Data extraction sheet.

Hospital name __________________________

Hospital city __________________________

Hospital state __________________________

Hospital country __________________________

Research network RIDEPLA

PERUKI

REPEM

PREDICT

PECARN

PEM-CRC

PERC

RISEUP-SPERG

PERQ

Notified of study via Research network

Social media

Title of guideline:

Date of guideline:

Source of guideline: Local Hospital

City

State

Country

Medical college

International Guideline

Referenced to: GINA

NICE

BTS / SIGN

National Heart, Lung, and Blood Institute (NHLBI)

State / regional guideline

Other

Language of guideline

English

Other (specify)

Translated by (initials):

Translation verified by (initials):

Data abstraction performed by (initials):

Does the asthma clinical guideline address:

Adults

School-aged children

Preschool children

Both adults and children

Not specified

How is asthma defined in the guideline?

(Free text response)

How are children defined in the guideline?

Are children grouped into different categories according to age (i.e. pre-schoolers, older children)?

If so, what are the age groups?

Age group #1

Age group #2

Age group #3

How is the assessment of asthma severity described in the guideline?

Is a scoring system recommended?

Which scoring system is recommended?

What are the “labels” and “thresholds” for each level of severity?

| Severity level | Label in guideline | Definition in guideline |
| --- | --- | --- |
| 1 (mildest) |  |  |
| 2 |  |  |
| 3 |  |  |
| 4 |  |  |
| 5 (most severe) |  |  |

What is the recommended initial treatment for severity level 1 (mildest) asthma?

Age group #1

Age group #2

Age group #3

What is the recommended initial treatment for severity level 2 asthma?

Age group #1

Age group #2

Age group #3

What is the recommended initial treatment for severity level 3 asthma?

Age group #1

Age group #2

Age group #3

What is the recommended initial treatment for severity level 4 asthma?

Age group #1

Age group #2

Age group #3

What is the recommended initial treatment for severity level 5 (most severe) asthma?

Age group #1

Age group #2

Age group #3

For children in age group #1, which of the following treatments are recommended?

If no recommendation, leave BLANK.

If recommended against (e.g. “Do not give”) then write “NO”

|  | Severity level 1 | Severity level 2 | Severity level 3 | Severity level 4 | Severity level 5 |
| --- | --- | --- | --- | --- | --- |
| Inhaled beta-2 agonists via puffer without spacer  (specify name of medication, dose, and dosing interval) |  |  |  |  |  |
| Inhaled beta-2 agonists via puffer and spacer  (specify name of medication, dose, and dosing interval) |  |  |  |  |  |
| Inhaled beta-2 agonists via nebuliser mask  (specify name of medication, dose, and dosing interval) |  |  |  |  |  |
| Oral beta-2 agonists  (specify name of medication, dose, and dosing interval) |  |  |  |  |  |
| Inhaled adrenaline via nebuliser mask  (specify name of medication, dose, and dosing interval) |  |  |  |  |  |
| Inhaled anticholinergic agent via puffer without spacer  (specify name of medication, dose, and dosing interval) |  |  |  |  |  |
| Inhaled anticholinergic agent via puffer and spacer  (specify name of medication, dose, and dosing interval) |  |  |  |  |  |
| Inhaled anticholinergic agent via nebuliser mask  (specify name of medication, dose, and dosing interval) |  |  |  |  |  |
| Oral anticholinergic agent  (specify name of medication, dose, and dosing interval) |  |  |  |  |  |
| Inhaled magnesium via nebuliser mask  (specify name of medication, dose, and dosing interval) |  |  |  |  |  |
| Inhaled heliox  (specify flow percentage used, and flow rate) |  |  |  |  |  |
| Low-flow nasal oxygen titrated to oxygen saturations  (specify maximum flow rate, and target oxygen saturation) |  |  |  |  |  |
| High-flow nasal oxygen titrated to oxygen saturations  (specify maximum flow rate, and target oxygen saturation) |  |  |  |  |  |
| Face-mask oxygen titrated to oxygen saturations  (specify maximum flow rate, type of mask, and target oxygen saturation) |  |  |  |  |  |
| Continuous positive airways pressure  (specify recommended settings) |  |  |  |  |  |
| Bi-level positive airways pressure  (specify recommended settings) |  |  |  |  |  |
| Subcutanous beta-2 agonist  (specify name of medication, dose, and dosing interval) |  |  |  |  |  |
| Subcutaneous adrenaline  (specify name of medication, dose, and dosing interval) |  |  |  |  |  |
| Intravenous magnesium – loading dose  (specify name of medication, dose, and dosing interval) |  |  |  |  |  |
| Intravenous magnesium – continuous infusion  (specify name of medication, dose, and dosing interval) |  |  |  |  |  |
| Intravenous beta-2 agonist  (specify name of medication, dose, and dosing interval) |  |  |  |  |  |
| Intravenous beta-2 agonist – continuous infusion  (specify name of medication, dose, and dosing interval) |  |  |  |  |  |
| Intravenous methylxanthine (theophylline / aminophylline)  (specify name of medication, dose, and dosing interval) |  |  |  |  |  |
| Intravenous methylxanthine (theophylline / aminophylline)– continuous infusion  (specify name of medication, dose, and dosing interval) |  |  |  |  |  |
| Oral corticosteroids  (specify name of medication, dose, and dosing interval) |  |  |  |  |  |
| Intravenous corticosteroids  (specify name of medication, dose, and dosing interval) |  |  |  |  |  |
| Intramuscular adrenaline  (specify name of medication, dose, and dosing interval) |  |  |  |  |  |
| Intravenous adrenaline infusion  (specify name of medication, dose, and dosing interval) |  |  |  |  |  |
| Intravenous ketamine  (specify name of medication, dose, and dosing interval) |  |  |  |  |  |
| Intravenous ketamine – continuous infusion  (specify name of medication, dose, and dosing interval) |  |  |  |  |  |

Recommendations for

- admission / discharge; intensive care referral; interhospital transfer
- medications at discharge
- follow-up management

For children in age group #2, which of the following treatments are recommended?

If no recommendation, leave BLANK.

If recommended against (e.g. “Do not give”) then write “NO”

|  | Severity level 1 | Severity level 2 | Severity level 3 | Severity level 4 | Severity level 5 |
| --- | --- | --- | --- | --- | --- |
| Inhaled beta-2 agonists via puffer without spacer  (specify name of medication, dose, and dosing interval) |  |  |  |  |  |
| Inhaled beta-2 agonists via puffer and spacer  (specify name of medication, dose, and dosing interval) |  |  |  |  |  |
| Inhaled beta-2 agonists via nebuliser mask  (specify name of medication, dose, and dosing interval) |  |  |  |  |  |
| Oral beta-2 agonists  (specify name of medication, dose, and dosing interval) |  |  |  |  |  |
| Inhaled adrenaline via nebuliser mask  (specify name of medication, dose, and dosing interval) |  |  |  |  |  |
| Inhaled anticholinergic agent via puffer without spacer  (specify name of medication, dose, and dosing interval) |  |  |  |  |  |
| Inhaled anticholinergic agent via puffer and spacer  (specify name of medication, dose, and dosing interval) |  |  |  |  |  |
| Inhaled anticholinergic agent via nebuliser mask  (specify name of medication, dose, and dosing interval) |  |  |  |  |  |
| Oral anticholinergic agent  (specify name of medication, dose, and dosing interval) |  |  |  |  |  |
| Inhaled magnesium via nebuliser mask  (specify name of medication, dose, and dosing interval) |  |  |  |  |  |
| Inhaled heliox  (specify flow percentage used, and flow rate) |  |  |  |  |  |
| Low-flow nasal oxygen titrated to oxygen saturations  (specify maximum flow rate, and target oxygen saturation) |  |  |  |  |  |
| High-flow nasal oxygen titrated to oxygen saturations  (specify maximum flow rate, and target oxygen saturation) |  |  |  |  |  |
| Face-mask oxygen titrated to oxygen saturations  (specify maximum flow rate, type of mask, and target oxygen saturation) |  |  |  |  |  |
| Continuous positive airways pressure  (specify recommended settings) |  |  |  |  |  |
| Bi-level positive airways pressure  (specify recommended settings) |  |  |  |  |  |
| Subcutanous beta-2 agonist  (specify name of medication, dose, and dosing interval) |  |  |  |  |  |
| Subcutaneous adrenaline  (specify name of medication, dose, and dosing interval) |  |  |  |  |  |
| Intravenous magnesium – loading dose  (specify name of medication, dose, and dosing interval) |  |  |  |  |  |
| Intravenous magnesium – continuous infusion  (specify name of medication, dose, and dosing interval) |  |  |  |  |  |
| Intravenous beta-2 agonist  (specify name of medication, dose, and dosing interval) |  |  |  |  |  |
| Intravenous beta-2 agonist – continuous infusion  (specify name of medication, dose, and dosing interval) |  |  |  |  |  |
| Intravenous methylxanthine (theophylline / aminophylline)  (specify name of medication, dose, and dosing interval) |  |  |  |  |  |
| Intravenous methylxanthine (theophylline / aminophylline)– continuous infusion  (specify name of medication, dose, and dosing interval) |  |  |  |  |  |
| Oral corticosteroids  (specify name of medication, dose, and dosing interval) |  |  |  |  |  |
| Intravenous corticosteroids  (specify name of medication, dose, and dosing interval) |  |  |  |  |  |
| Intramuscular adrenaline  (specify name of medication, dose, and dosing interval) |  |  |  |  |  |
| Intravenous adrenaline infusion  (specify name of medication, dose, and dosing interval) |  |  |  |  |  |
| Intravenous ketamine  (specify name of medication, dose, and dosing interval) |  |  |  |  |  |
| Intravenous ketamine – continuous infusion  (specify name of medication, dose, and dosing interval) |  |  |  |  |  |

Recommendations for

- admission / discharge; intensive care referral; inter hospital transfer
- medications at discharge
- follow-up management

For children in age group #3, which of the following treatments are recommended?

If no recommendation, leave BLANK.

If recommended against (e.g. “Do not give”) then write “NO”

|  | Severity level 1 | Severity level 2 | Severity level 3 | Severity level 4 | Severity level 5 |
| --- | --- | --- | --- | --- | --- |
| Inhaled beta-2 agonists via puffer without spacer  (specify name of medication, dose, and dosing interval) |  |  |  |  |  |
| Inhaled beta-2 agonists via puffer and spacer  (specify name of medication, dose, and dosing interval) |  |  |  |  |  |
| Inhaled beta-2 agonists via nebuliser mask  (specify name of medication, dose, and dosing interval) |  |  |  |  |  |
| Oral beta-2 agonists  (specify name of medication, dose, and dosing interval) |  |  |  |  |  |
| Inhaled adrenaline via nebuliser mask  (specify name of medication, dose, and dosing interval) |  |  |  |  |  |
| Inhaled anticholinergic agent via puffer without spacer  (specify name of medication, dose, and dosing interval) |  |  |  |  |  |
| Inhaled anticholinergic agent via puffer and spacer  (specify name of medication, dose, and dosing interval) |  |  |  |  |  |
| Inhaled anticholinergic agent via nebuliser mask  (specify name of medication, dose, and dosing interval) |  |  |  |  |  |
| Oral anticholinergic agent  (specify name of medication, dose, and dosing interval) |  |  |  |  |  |
| Inhaled magnesium via nebuliser mask  (specify name of medication, dose, and dosing interval) |  |  |  |  |  |
| Inhaled heliox  (specify flow percentage used, and flow rate) |  |  |  |  |  |
| Low-flow nasal oxygen titrated to oxygen saturations  (specify maximum flow rate, and target oxygen saturation) |  |  |  |  |  |
| High-flow nasal oxygen titrated to oxygen saturations  (specify maximum flow rate, and target oxygen saturation) |  |  |  |  |  |
| Face-mask oxygen titrated to oxygen saturations  (specify maximum flow rate, type of mask, and target oxygen saturation) |  |  |  |  |  |
| Continuous positive airways pressure  (specify recommended settings) |  |  |  |  |  |
| Bi-level positive airways pressure  (specify recommended settings) |  |  |  |  |  |
| Subcutanous beta-2 agonist  (specify name of medication, dose, and dosing interval) |  |  |  |  |  |
| Subcutaneous adrenaline  (specify name of medication, dose, and dosing interval) |  |  |  |  |  |
| Intravenous magnesium – loading dose  (specify name of medication, dose, and dosing interval) |  |  |  |  |  |
| Intravenous magnesium – continuous infusion  (specify name of medication, dose, and dosing interval) |  |  |  |  |  |
| Intravenous beta-2 agonist  (specify name of medication, dose, and dosing interval) |  |  |  |  |  |
| Intravenous beta-2 agonist – continuous infusion  (specify name of medication, dose, and dosing interval) |  |  |  |  |  |
| Intravenous methylxanthine (theophylline / aminophylline)  (specify name of medication, dose, and dosing interval) |  |  |  |  |  |
| Intravenous methylxanthine (theophylline / aminophylline)– continuous infusion  (specify name of medication, dose, and dosing interval) |  |  |  |  |  |
| Oral corticosteroids  (specify name of medication, dose, and dosing interval) |  |  |  |  |  |
| Intravenous corticosteroids  (specify name of medication, dose, and dosing interval) |  |  |  |  |  |
| Intramuscular adrenaline  (specify name of medication, dose, and dosing interval) |  |  |  |  |  |
| Intravenous adrenaline infusion  (specify name of medication, dose, and dosing interval) |  |  |  |  |  |
| Intravenous ketamine  (specify name of medication, dose, and dosing interval) |  |  |  |  |  |
| Intravenous ketamine – continuous infusion  (specify name of medication, dose, and dosing interval) |  |  |  |  |  |

Recommendations for

- admission / discharge; intensive care referral; inter hospital transfer
- medications at discharge
- follow-up management
