# Supplementary material for: Acute severe paediatric asthma: study protocol for the development of a core outcome set, a Pediatric Emergency Reserarch Networks (PERN) study
Source: Trials. 2020 Jan 13;21:72. doi: 10.1186/s13063-019-3785-6 (PMC6956506; doi:10.1186/s13063-019-3785-6)
Supplement: Supplementary file 4 — Additional file 4. SPIRIT 2013 Checklist: Recommended items to address in a clinical trial protocol and related documents. [file 13063_2019_3785_MOESM4_ESM.doc]

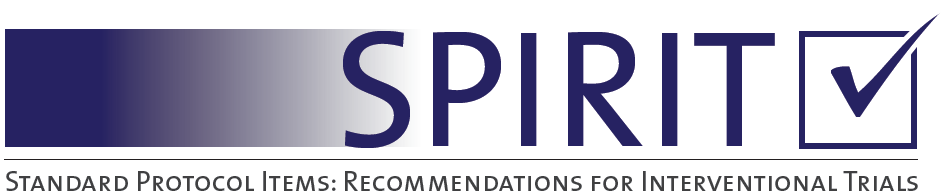


SPIRIT 2013 Checklist: Recommended items to address in a clinical trial protocol and related documents*

| Section/item | ItemNo | Description |
| --- | --- | --- |
| **Administrative information** | | |
| Title | 1 | Descriptive title identifying the study design, population, interventions, and, if applicable, trial acronym  **Title describes study design** |
| Trial registration | 2a | Trial identifier and registry name. If not yet registered, name of intended registry  **The trial is registered with the COMET initiative (top paragraph of page 15)**  **The systematic review is registered with PROSPERO (page 16)**  **The Cochrane Overview is registered with the Cochrane Collaboration** |
|  | 2b | All items from the World Health Organization Trial Registration Data Set  **N/A: The paper does not describe a clinical trial.** |
| Protocol version | 3 | Date and version identifier  **This is a protocol to describe multiple planned studies. The version submitted is current as of July 2019. (top paragraph of page 15)** |
| Funding | 4 | Sources and types of financial, material, and other support  **Acknowledgements section (page 31)** |
| Roles and responsibilities | 5a | Names, affiliations, and roles of protocol contributors  **Authors are listed from pages 1-7. Roles are listed on page 8** |
|  | 5b | Name and contact information for the trial sponsor  **N/A: The paper does not describe a clinical trial.** |
|  | 5c | Role of study sponsor and funders, if any, in study design; collection, management, analysis, and interpretation of data; writing of the report; and the decision to submit the report for publication, including whether they will have ultimate authority over any of these activities  **See statement under acknowledgements (page 31).** |
|  | 5d | Composition, roles, and responsibilities of the coordinating centre, steering committee, endpoint adjudication committee, data management team, and other individuals or groups overseeing the trial, if applicable (see Item 21a for data monitoring committee)  **N/A: The paper does not describe a clinical trial.** |
| Introduction |  |  |
| Background and rationale | 6a | Description of research question and justification for undertaking the trial, including summary of relevant studies (published and unpublished) examining benefits and harms for each intervention  **Page 11-14** |
|  | 6b | Explanation for choice of comparators  **N/A: The paper does not describe a clinical trial.** |
| Objectives | 7 | Specific objectives or hypotheses  **Page 14-15** |
| Trial design | 8 | Description of trial design including type of trial (eg, parallel group, crossover, factorial, single group), allocation ratio, and framework (eg, superiority, equivalence, noninferiority, exploratory)  **This is a protocol to describe multiple planned studies. An overview of the study types and planned methods is provided on pages 15-16** |
| Methods: Participants, interventions, and outcomes | | |
| Study setting | 9 | Description of study settings (eg, community clinic, academic hospital) and list of countries where data will be collected. Reference to where list of study sites can be obtained  **Interview studies: pages 17-18**  **Delphi study – page 20**  **Retrospective study – page 26** |
| Eligibility criteria | 10 | Inclusion and exclusion criteria for participants. If applicable, eligibility criteria for study centres and individuals who will perform the interventions (eg, surgeons, psychotherapists)  **Interview studies: top of page 18**  **Delphi study – page 20**  **Retrospective study – page 27** |
| Interventions | 11a | Interventions for each group with sufficient detail to allow replication, including how and when they will be administered  **N/A: The paper does not describe a clinical trial.** |
|  | 11b | Criteria for discontinuing or modifying allocated interventions for a given trial participant (eg, drug dose change in response to harms, participant request, or improving/worsening disease)  **N/A: The paper does not describe a clinical trial.** |
|  | 11c | Strategies to improve adherence to intervention protocols, and any procedures for monitoring adherence (eg, drug tablet return, laboratory tests)  **N/A: The paper does not describe a clinical trial.** |
|  | 11d | Relevant concomitant care and interventions that are permitted or prohibited during the trial  **N/A: The paper does not describe a clinical trial.** |
| Outcomes | 12 | Primary, secondary, and other outcomes, including the specific measurement variable (eg, systolic blood pressure), analysis metric (eg, change from baseline, final value, time to event), method of aggregation (eg, median, proportion), and time point for each outcome. Explanation of the clinical relevance of chosen efficacy and harm outcomes is strongly recommended  Described for each project as follows:  **Delphi study – p22**  **Clinical practice guidelines – p24-26**  **Retrospective study – p27** |
| Participant timeline | 13 | Time schedule of enrolment, interventions (including any run-ins and washouts), assessments, and visits for participants. A schematic diagram is highly recommended (see Figure)  **N/A: The paper does not describe a clinical trial.** |
| Sample size | 14 | Estimated number of participants needed to achieve study objectives and how it was determined, including clinical and statistical assumptions supporting any sample size calculations  **Page 19 discusses sample size for the qualitative studies.**  **Page 29 discusses sample size calculations for the retrospective study/** |
| Recruitment | 15 | Strategies for achieving adequate participant enrolment to reach target sample size  **Page 20 (for Delphi study)** |
| **Methods: Assignment of interventions (for controlled trials)** | | |
| Allocation: |  |  |
| Sequence generation | 16a | Method of generating the allocation sequence (eg, computer-generated random numbers), and list of any factors for stratification. To reduce predictability of a random sequence, details of any planned restriction (eg, blocking) should be provided in a separate document that is unavailable to those who enrol participants or assign interventions  **The paper does not describe a clinical trial.**  **If >1000 records are identified at a particular site for the retrospective study, patients will be randomly selected (see page 29 for details)** |
| Allocation concealment mechanism | 16b | Mechanism of implementing the allocation sequence (eg, central telephone; sequentially numbered, opaque, sealed envelopes), describing any steps to conceal the sequence until interventions are assigned  **N/A: The paper does not describe a clinical trial.** |
| Implementation | 16c | Who will generate the allocation sequence, who will enrol participants, and who will assign participants to interventions  **N/A: The paper does not describe a clinical trial.** |
| Blinding (masking) | 17a | Who will be blinded after assignment to interventions (eg, trial participants, care providers, outcome assessors, data analysts), and how  **N/A: The paper does not describe a clinical trial.** |
|  | 17b | If blinded, circumstances under which unblinding is permissible, and procedure for revealing a participant’s allocated intervention during the trial  **N/A: The paper does not describe a clinical trial.** |
| **Methods: Data collection, management, and analysis** | | |
| Data collection methods | 18a | Plans for assessment and collection of outcome, baseline, and other trial data, including any related processes to promote data quality (eg, duplicate measurements, training of assessors) and a description of study instruments (eg, questionnaires, laboratory tests) along with their reliability and validity, if known. Reference to where data collection forms can be found, if not in the protocol  **Systematic review – p16**  **Clinical guidelines study – p24-26**  **Retrospective chart review – p27** |
|  | 18b | Plans to promote participant retention and complete follow-up, including list of any outcome data to be collected for participants who discontinue or deviate from intervention protocols  **The paper does not describe a clinical trial.**  **We will provide clear guidance and reminders to the participants in the Delphi study (page 20-21)** |
| Data management | 19 | Plans for data entry, coding, security, and storage, including any related processes to promote data quality (eg, double data entry; range checks for data values). Reference to where details of data management procedures can be found, if not in the protocol  **Clinical guidelines study – p24-26**  **Retrospective study – p27-28** |
| Statistical methods | 20a | Statistical methods for analysing primary and secondary outcomes. Reference to where other details of the statistical analysis plan can be found, if not in the protocol  **Retrospective study – p28** |
|  | 20b | Methods for any additional analyses (eg, subgroup and adjusted analyses)  **Qualitative study – p19**  **Exploratory data analysis (retrospective study) – p27** |
|  | 20c | Definition of analysis population relating to protocol non-adherence (eg, as randomised analysis), and any statistical methods to handle missing data (eg, multiple imputation)  **N/A: The paper does not describe a clinical trial.** |
| **Methods: Monitoring** | | |
| Data monitoring | 21a | Composition of data monitoring committee (DMC); summary of its role and reporting structure; statement of whether it is independent from the sponsor and competing interests; and reference to where further details about its charter can be found, if not in the protocol. Alternatively, an explanation of why a DMC is not needed  **N/A: The paper does not describe a clinical trial.** |
|  | 21b | Description of any interim analyses and stopping guidelines, including who will have access to these interim results and make the final decision to terminate the trial  **N/A: The paper does not describe a clinical trial.** |
| Harms | 22 | Plans for collecting, assessing, reporting, and managing solicited and spontaneously reported adverse events and other unintended effects of trial interventions or trial conduct  **N/A: The paper does not describe a clinical trial.** |
| Auditing | 23 | Frequency and procedures for auditing trial conduct, if any, and whether the process will be independent from investigators and the sponsor  **N/A: The paper does not describe a clinical trial.** |
| Ethics and dissemination | | |
| Research ethics approval | 24 | Plans for seeking research ethics committee/institutional review board (REC/IRB) approval  **Page 20.** |
| Protocol amendments | 25 | Plans for communicating important protocol modifications (eg, changes to eligibility criteria, outcomes, analyses) to relevant parties (eg, investigators, REC/IRBs, trial participants, trial registries, journals, regulators)  **N/A: The paper does not describe a clinical trial.** |
| Consent or assent | 26a | Who will obtain informed consent or assent from potential trial participants or authorised surrogates, and how (see Item 32)  **N/A: The paper does not describe a clinical trial.** |
|  | 26b | Additional consent provisions for collection and use of participant data and biological specimens in ancillary studies, if applicable  **N/A: The paper does not describe a clinical trial.** |
| Confidentiality | 27 | How personal information about potential and enrolled participants will be collected, shared, and maintained in order to protect confidentiality before, during, and after the trial  **Interview study data will be de-identified – p19**  **Retrospective study datat will be de-identified – p27** |
| Declaration of interests | 28 | Financial and other competing interests for principal investigators for the overall trial and each study site  **Page 8 – none of the authors declare any competing interests.** |
| Access to data | 29 | Statement of who will have access to the final trial dataset, and disclosure of contractual agreements that limit such access for investigators  **Page 27-28 – statement on access to data.** |
| Ancillary and post-trial care | 30 | Provisions, if any, for ancillary and post-trial care, and for compensation to those who suffer harm from trial participation  **N/A: The paper does not describe a clinical trial.** |
| Dissemination policy | 31a | Plans for investigators and sponsor to communicate trial results to participants, healthcare professionals, the public, and other relevant groups (eg, via publication, reporting in results databases, or other data sharing arrangements), including any publication restrictions  **Page 31 – we aim to disseminate our results through the usual scientific channels (scientific meetings and journal publication).** |
|  | 31b | Authorship eligibility guidelines and any intended use of professional writers  **See statement on page 31** |
|  | 31c | Plans, if any, for granting public access to the full protocol, participant-level dataset, and statistical code  **N/A: The paper does not describe a clinical trial. Publication of this manuscript will enable public access to the study protocol.** |
| Appendices |  |  |
| Informed consent materials | 32 | Model consent form and other related documentation given to participants and authorised surrogates  **N/A: The paper does not describe a clinical trial.** |
| Biological specimens | 33 | Plans for collection, laboratory evaluation, and storage of biological specimens for genetic or molecular analysis in the current trial and for future use in ancillary studies, if applicable  **N/A: The paper does not describe a clinical trial.** |

*It is strongly recommended that this checklist be read in conjunction with the SPIRIT 2013 Explanation & Elaboration for important clarification on the items. Amendments to the protocol should be tracked and dated. The SPIRIT checklist is copyrighted by the SPIRIT Group under the Creative Commons “[Attribution-NonCommercial-NoDerivs 3.0 Unported](http://www.creativecommons.org/licenses/by-nc-nd/3.0/)” license.
